# Looking back…….looking ahead

**DOI:** 10.4103/0972-9941.62524

**Published:** 2010

**Authors:** Tehemton E Udwadia

**Affiliations:** Editor-in-Chief, JMAS, Department of Minimal Access Surgery, P D Hinduja National Hospital, Breach Candy and Parsee General Hospitals, Veer Savarkar Marg, Mahim, Mumbai - 400 016, India

The Journal of Minimal Access Surgery (JMAS), the publication of the Indian Association of Gastrointestinal Endo-Surgeons completes five years of publication. The Editorial Board reviews the first five years of JMAS and tries to take stock and analyse how it has coped with its numerous difficulties, how it could have coped better, to asses what has been achieved and where we have fallen short and to reiterate the priorities, goals and ambitions set out at the launch of the Journal. Above all, with our learning curve over the last five years, to use the experience to regroup, summon up all our resources and strive with greater determination and commitment to raise the Journal to a level befitting the growth of minimal access surgery (MAS) in India.

The IAGES Executive Committee at its meeting in February 2004 conceived the idea of starting its own Journal, the JMAS. Funded with a seed money donation of Rs. 60,000 the Journal has willy-nilly kept its head above water inspite of escalating costs by cutting corners yet gaining strength and confidence on its own resources. It would help our growth greatly (every extra article published needs greater financial support) if conference and workshop activities were advertised in the Journal and a small part of the surplus generated by these activities be diverted to the Journal.

The IAGES is a democratic transparent society and it is the policy of the Journal to follow the principles of its parent association. JMAS provides immediate free access to the full text of the articles. It also permits authors' self-archiving. The JMAS does not charge article submission, processing or publication fee from the authors or authors' institution. The open access policy of the JMAS aims at increasing the visibility and accessibility of the published content and thus providing the desirable research impact and author exposure. JMAS has an easy to use electronic manuscript submission system eliminating use of postal or hard copy submission. Online submission and processing of articles has resulted in a decrease in the submission to decision time.

Including three special issues, the JMAS, a quarterly Journal, has had 20 issues over the last 5 years. To maintain quality, even at the cost of publishing fewer articles and some incumbent delay in processing, there is a rigorous peer-review process that results in rejection of over 55% manuscripts over the years. With this rejection rate, it is mandatory that we receive far more manuscripts to bring out a healthy size issue each time. While pleased that about 40% of all manuscripts we receive are from out of India (which suffer the same rejection rate), our great concern is the need to have more manuscripts generated from the immense MAS workload in our country.

Journal performance needs improvement on two scores – decreasing the submission to acceptance to publication time and bringing out each issue exactly on time. Both areas of default are interrelated, and the common drag factor is an undue time the manuscript is under the review process – over 50% of the total submissions to acceptance time. Over the last 5 years, JMAS is indexed in 22 databases like Bioline International, Google Scholar, Pubmed and Pubmed Central. Getting indexed with Science Citation Index is our ultimate goal. Our website continues to have a large and wide readership – over 172 countries and 6,734 cities, with the maximum visits from London and New York. It is fortunate that Dr. D. S. Bhandarkar, one of the Editors, has oversize wide shoulders –he has carried the great part of the Journal over the last 5 years. Equally vital to JMAS is our publisher Dr. D. K. Sahu of Medknow Publications who has worked tirelessly to maintain standards and push for Indexing.

To look ahead, one can shorten the review time, streamline functioning, scrape together more revenue and keep quality control, but the only real power-generator of any Journal is the number and quality of the manuscripts it receives. India is in the very forefront of MAS, both in terms of quality or results, quantity of patients, variety of procedures and path-breaking innovations. Our members have been major forces in the genesis and growth of Natural Orifice Translumenal Endoscopic Surgery and Single Incision Laparoscopic Surgery. The Indian surgeon who performs MAS from the large cities to the smallest town, working with the utmost sophistication in instrumentation to using atmospheric air to create pneumoperitoneum (to the dismay of the purist!), each one in every part of India is the pride of the country. Can we transfer this pride beyond individuals or Departments to pride in the overall structure of Indian MAS – a pride best translated in showcasing our problems, advances and achievements in MAS in our own Journal? This then is the future thrust of JMAS – to invoke a sense of proprietary pride in our Journal so that surgeons practising MAS proffer their best work to their own Journal. And, this plea goes beyond IAGES Members to Members of all MAS Societies who share our fervour and to the many thousands more who are not members of any Society but a large part of our MAS thrust.

Through hiccups and stumbles, the JMAS has more than survived. It has grown over five years, and if success is measured by the obstacles overcome while trying to succeed, it has achieved some measure of success. As the only English language MAS Journal in the entire Asia-Pacific region, it needs to do much more to project and raise the level of MAS in this region and to spread MAS growth in the vast and most populous part of the developing world. The Editorial Board will strive to fulfil its mission, achieve its dream, firm in its belief that “the future belongs to those who believe in the beauty of their dreams”.

